# Corrected Four-Sphere Head Model for EEG Signals

**DOI:** 10.3389/fnhum.2017.00490

**Published:** 2017-10-18

**Authors:** Solveig Næss, Chaitanya Chintaluri, Torbjørn V. Ness, Anders M. Dale, Gaute T. Einevoll, Daniel K. Wójcik

**Affiliations:** ^1^Department of Informatics, University of Oslo, Oslo, Norway; ^2^Department of Neurophysiology, Nencki Institute of Experimental Biology, Warsaw, Poland; ^3^Faculty of Science and Technology, Norwegian University of Life Sciences, Ås, Norway; ^4^Departments of Neurosciences and Radiology, University of California, San Diego, La Jolla, CA, United States; ^5^Department of Physics, University of Oslo, Oslo, Norway

**Keywords:** four-sphere model, head model, EEG, dipole source, LFP, FEM

## Abstract

The EEG signal is generated by electrical brain cell activity, often described in terms of current dipoles. By applying EEG forward models we can compute the contribution from such dipoles to the electrical potential recorded by EEG electrodes. Forward models are key both for generating understanding and intuition about the neural origin of EEG signals as well as inverse modeling, i.e., the estimation of the underlying dipole sources from recorded EEG signals. Different models of varying complexity and biological detail are used in the field. One such analytical model is the *four-sphere model* which assumes a four-layered spherical head where the layers represent brain tissue, cerebrospinal fluid (CSF), skull, and scalp, respectively. While conceptually clear, the mathematical expression for the electric potentials in the four-sphere model is cumbersome, and we observed that the formulas presented in the literature contain errors. Here, we derive and present the correct analytical formulas with a detailed derivation. A useful application of the analytical four-sphere model is that it can serve as ground truth to test the accuracy of numerical schemes such as the Finite Element Method (FEM). We performed FEM simulations of the four-sphere head model and showed that they were consistent with the corrected analytical formulas. For future reference we provide scripts for computing EEG potentials with the four-sphere model, both by means of the correct analytical formulas and numerical FEM simulations.

## 1. Introduction

Electroencephalography (EEG), that is, the recording of electrical potentials at the scalp, has been of key importance for probing human brain activity for more than half a century (Nunez and Srinivasan, 2006; Schomer and da Silva, 2012). The EEG signal is generated by current dipoles set up by transmembrane currents in brain cells, and EEG *forward models* aim to compute the contribution from such current dipoles to the electrical potential recorded by EEG electrodes (Hämäläinen et al., 1993; Sanei and Chambers, 2007). Forward models are useful for generating understanding and intuition about the neural origin of EEG signals. They are also key for inverse modeling, i.e., the estimation of the underlying sources based on recorded EEG signals, and for generation of benchmarking data against which candidate methods for EEG data analysis methods and simulation schemes for EEG can be tested.

While the link between the current sources and the resulting potentials in principle is well described by volume-conductor theory, the practical application of this theory is not easy because the cortical tissue, the cerebrospinal fluid (CSF), the skull, and the scalp, all have different electrical conductivities (Nunez and Srinivasan, 2006).

Different forward modeling schemes approximate the geometries and conductivities of the head with various levels of biological detail. On one side we have the spherical head models that can provide analytical formulas for the EEG potentials generated by current dipoles. At the other side of the spectrum we have numerically comprehensive forward modeling schemes, including realistic geometries and electrical conductivities, even electrically anisotropic tissue (Bangera et al., 2010; Vorwerk et al., 2014). These different forward models come with their different advantages and disadvantages in terms of speed, accuracy and interpretability of results (De Munck et al., 2012).

In this paper, we address the four-sphere head model where the head is modeled as four concentric spherical layers. Here, the four layers represent brain tissue, CSF, skull, and scalp. The Poisson equation, which describes the electric fields of the brain within volume-conductor theory, is solved for each layer separately, and the mathematical solutions are matched at the layer interfaces to obtain an analytical expression for the EEG signal as set up by a current source in the brain tissue. The relatively small number of parameters makes the four-sphere model an obvious candidate for exploring and gaining intuition about the nature of EEG signals. Since the solution is analytical and requires little computation time compared to complex numerical schemes, it can be used to quickly test analysis methods and hypotheses. The most popular version of the four-sphere model was presented in Srinivasan et al. (1998); and later in the classic EEG reference book Electric Fields of the Brain (Nunez and Srinivasan, 2006). This model has been used to generate benchmarking data for testing of EEG signal analysis methods, (e.g., Wong et al., 2008; Chu et al., 2012; Peraza et al., 2012), and it is also useful for validation of more general and numerically comprehensive numerical schemes such as the Boundary Element Method (BEM) (Brebbia et al., 2012) and the Finite Element Method (FEM) (Larson and Bengzon, 2013). The FEM approach is the most general and can, in principle, take into account an arbitrarily complicated spatial distribution of electrical conductivity representing the electrical properties of the head (Bangera et al., 2010; Huang et al., 2016). This is done by building a numerical mesh for the head model with the electrical conductivity specified at each mesh point. The mesh construction is a research problem by itself and several mesh-generation tools are available, which often provide slightly different results (Geuzaine, 2009; Kehlet, 2016). The analytical solution for the four-sphere model can serve as a ground truth for testing of different numerical schemes.

While conceptually clear, the mathematical expression of the four-sphere forward model is quite involved and rederiving the expression we discovered errors in the formulas both in the original paper and in the book. Due to the importance of the four-sphere model, we here derive and provide the correct analytical formulas for future reference. We tested our formulas by verifying that the solutions for neighboring layers matched on the layer boundaries. Moreover, when the conductivities for all the layers in the model were set to the same value, the model reduced to the well-known homogeneous single-sphere model as it should. We also verified that the model solution reduces to the formula for the extracellular potential from a current dipole in an infinite homogeneous space, when the layer radii go to infinity and the conductivities for all model layers are equal (not shown). As an application, we performed FEM simulations of the four-sphere model which were consistent with the corrected analytical formulas.

## 2. Methods

### 2.1. Four-sphere model

**Figure 1:**
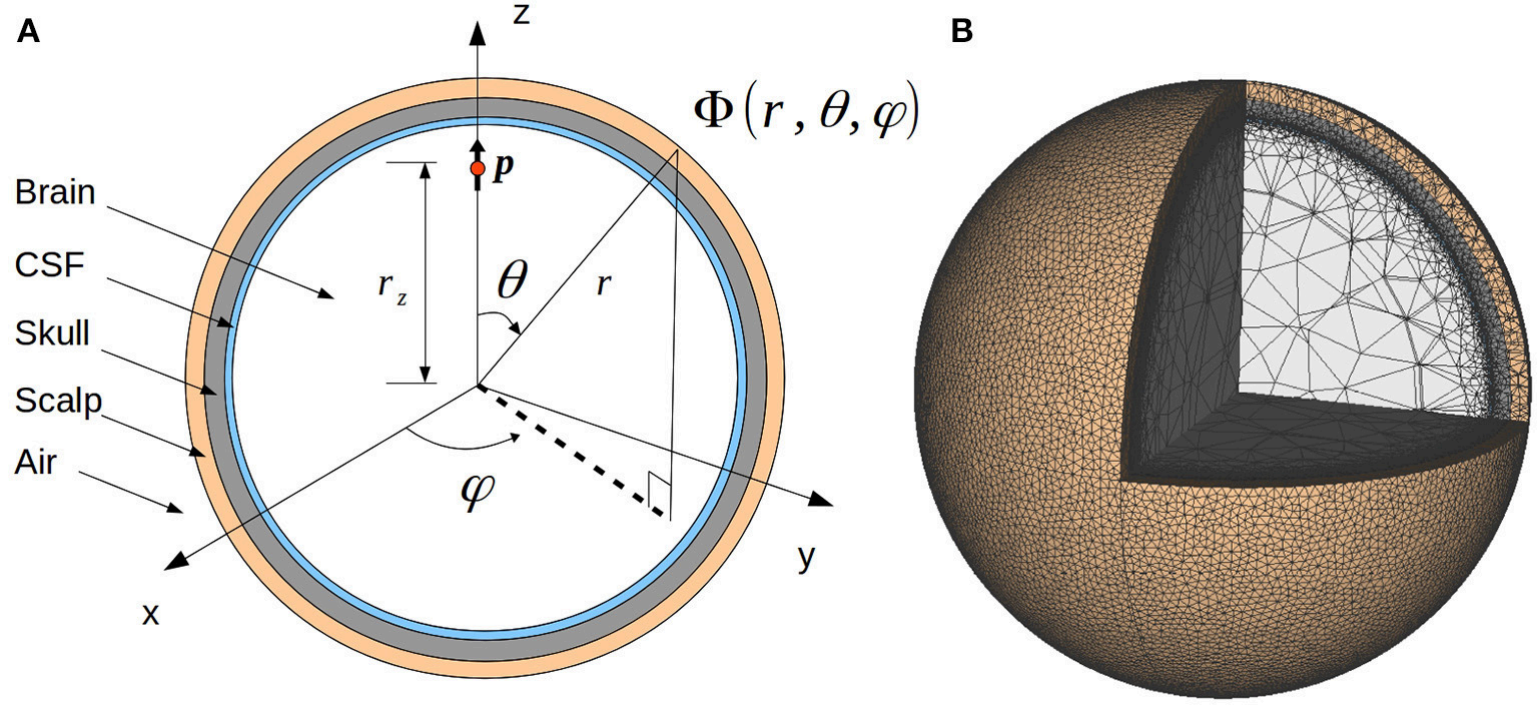
Illustration of the four-sphere head model. **(A)** Cross-section of the four-sphere head model, with the different colors corresponding to the different head layers: brain, CSF, skull, and scalp. The current dipole *p* is located in the brain layer, at a distance *r*_*z*_ from the center of the sphere. In all the subsequent figures, the dipole is placed in the *x* = 0 plane, at the z-axis (*r*_*z*_ = 7.8 cm). **(B)** Mesh of the four-sphere model used in the FEM simulations illustrating the different electrical conductivity values for each of the spheres.

By assuming the quasi-static approximation of Maxwell's equations and using the well-established volume-conductor theory, the electric potential Φ can be found by solving the Poisson equation (Nunez and Srinivasan, 2006),

(1) $$\begin{array}{r} \nabla \cdot \sigma \left(\text{r}\right)\nabla \Phi \left(\text{r},t\right)=-C\left(\text{r},t\right), \end{array}$$

where *C*(**r**, *t*) is the density of current sources. σ(**r**) is the position-dependent conductivity of the medium, here assumed to be isotropic so that σ(**r**) is a scalar. The four-sphere model is a specific solution of this equation which assumes that the conductive medium consists of four spherical layers representing specific constituents of the head: brain tissue, CSF, skull, and scalp (Figure 1A). In the computations below, these layers are labeled by *s* = 1 to 4, respectively. The conductivity σ_*s*_(**r**) is assumed to be homogeneous, i.e., constant within each layer and independent of frequency (Pettersen et al., 2012). In the examples below we assume the same values of conductivities and concentric shell radii as in Nunez and Srinivasan (2006), see Table 1. The solution of Equation (1) is subject to the following boundary conditions (where *s* = 1, 2, 3), assuring continuity of both electrical potential and current across the layer boundaries, and no current escaping the outer layer (Nunez and Srinivasan, 2006):

(2) $$\begin{array}{r} \Phi^{s+1}\left(r_{s}\right)=\Phi^{s}\left(r_{s}\right) \end{array}$$

(3) $$\begin{array}{r} \sigma_{s+1}\frac{\partial \Phi^{s+1}}{\partial r}\left(r_{s}\right)=\sigma_{s}\frac{\partial \Phi^{s}}{\partial r}\left(r_{s}\right) \end{array}$$

(4) $$\begin{array}{r} \frac{\partial \Phi^{4}}{\partial r}\left(r_{4}\right)=0. \end{array}$$

**Table 1:** Radii and electrical conductivities of the present four-sphere model.

**Labels**	**Name**	**Radius (cm)**	**σ (S/m)**
1	Brain	7.9	σ_brain_ = 0.33
2	CSF	8.0	5σ_brain_
3	Skull	8.5	σ_brain_/^*K*^
4	Scalp	9.0	σ_brain_

*σ is the conductivity in each of the specified regions. Three variants of the model were considered with skull conductivity reduced by a factor K (20, 40, or 80) compared to the conductivity of the brain*.

### 2.2. Analytical solution of the four-sphere head model

The solution of Equation (1) takes different forms for tangential and radial dipoles, and any dipole can be decomposed into a linear combination of these two. The following derivations are based on Appendix G and H in Nunez and Srinivasan (2006), and are described in more detail in Appendix 1 in Supplementary Materials.

#### 2.2.1. Radial dipole

Nunez and Srinivasan (2006) give the following equations for calculating extracellular potentials from a radial dipole in the four sphere model. The potential in the inner sphere, the brain, is given by Φ^1^(*r*, θ), while Φ^*s*^(*r*, θ) gives the potential in CSF, skull, and scalp, for *s* = 2, 3, 4, respectively,

(5) $$\begin{array}{r} \Phi^{1}\left(r,\theta \right)=\frac{p}{4\pi \sigma_{1}r_{z}^{2}}\overset{\infty }{\underset{n\;=\;1}{\sum }}\left[A_{n}^{1}{\left(\frac{r}{r_{1}}\right)}^{n}+{\left(\frac{r_{z}}{r}\right)}^{n+1}\right]nP_{n}\left(\cos\theta \right)\\ r_{z}<r\leq r_{1}, \end{array}$$

(6) $$\begin{array}{r} \Phi^{s}\left(r,\theta \right)=\frac{p}{4\pi \sigma_{1}r_{z}^{2}}\overset{\infty }{\underset{n\;=\;1}{\sum }}\left[A_{n}^{s}{\left(\frac{r}{r_{s}}\right)}^{n}+B_{n}^{s}{\left(\frac{r_{s}}{r}\right)}^{n+1}\right]nP_{n}\left(\cos\theta \right)\\ r_{s-1}\leq r\leq r_{s}. \end{array}$$

Here, Φ^*s*^ is the extracellular potential measured at radius *r* in shell number *s*, of external radius *r*_*s*_, from current dipole moment with magnitude *p* at radial location *r*_*z*_. The conductivity of sphere *s* is denoted by σ_*s*_, $A_{n}^{s}$ and $B_{n}^{s}$ are coefficients depending on the shell radii and conductivities, and *P*_*n*_(cosθ) is the *n*-th Legendre Polynomial where θ is the angle between measurement and dipole location vectors. From the boundary conditions listed in Equations (2)–(4), we can compute $A_{n}^{s}$, for *s* = 1, 2, 3, 4 and $B_{n}^{s}$, for *s* = 2, 3, 4, using the notation σ_*ij*_ ≡ σ_*i*_/σ_*j*_ and *r*_*ij*_ ≡ *r*_*i*_/*r*_*j*_:

(7) $$\begin{array}{r} A_{n}^{1}=\frac{\frac{n+1}{n}\sigma_{12}+Z_{n}}{\sigma_{12}-Z_{n}}r_{z1}^{n+1} \end{array}$$

(8) $$\begin{array}{r} A_{n}^{2}=\frac{A_{n}^{1}+r_{z1}^{n+1}}{r_{12}^{n}+r_{21}^{n+1}Y_{n}} \end{array}$$

(9) $$\begin{array}{r} B_{n}^{2}=Y_{n}A_{n}^{2} \end{array}$$

(10) $$\begin{array}{r} A_{n}^{3}=\frac{A_{n}^{2}+B_{n}^{2}}{r_{23}^{n}+r_{32}^{n+1}V_{n}} \end{array}$$

(11) $$\begin{array}{r} B_{n}^{3}=V_{n}A_{n}^{3} \end{array}$$

(12) $$\begin{array}{r} A_{n}^{4}=\frac{n+1}{n}\frac{A_{n}^{3}+B_{n}^{3}}{\frac{n+1}{n}r_{34}^{n}+r_{43}^{n+1}} \end{array}$$

(13) $$\begin{array}{r} B_{n}^{4}=\frac{n}{n+1}A_{n}^{4} \end{array}$$

(14) $$\begin{array}{r} V_{n}=\frac{\frac{n}{n+1}\sigma_{34}-\frac{r_{34}^{n}-r_{43}^{n+1}}{\frac{n+1}{n}r_{34}^{n}+r_{43}^{n+1}}}{\sigma_{34}+\frac{r_{34}^{n}-r_{43}^{n+1}}{\frac{n+1}{n}r_{34}^{n}+r_{43}^{n+1}}} \end{array}$$

(15) $$\begin{array}{r} Y_{n}=\frac{\frac{n}{n+1}\sigma_{23}-\frac{\frac{n}{n+1}r_{23}^{n}-V_{n}r_{32}^{n+1}}{r_{23}^{n}+V_{n}r_{32}^{n+1}}}{\sigma_{23}+\frac{\frac{n}{n+1}r_{23}^{n}-V_{n}r_{32}^{n+1}}{r_{23}^{n}+V_{n}r_{32}^{n+1}}} \end{array}$$

(16) $$\begin{array}{r} Z_{n}=\frac{r_{12}^{n}-\frac{n+1}{n}Y_{n}r_{21}^{n+1}}{r_{12}^{n}+Y_{n}r_{21}^{n+1}}. \end{array}$$

Equations (5) and (6) are in accordance with Equations (G.1.9–10) in Appendix G of Nunez and Srinivasan (2006) and Equation (A–1) in Srinivasan et al. (1998), Appendix A. However, some of the above coefficients [Equations (7)–(16)] are different from the ones given in Nunez and Srinivasan (2006) and Srinivasan et al. (1998), see Appendix 1 in Supplementary Materials for specifics.

#### 2.2.2. Tangential dipole

The extracellular potential from a tangential dipole in a concentric-shells model is given by Equation (H.2.1) in Appendix H of Nunez and Srinivasan (2006), and takes the following form:

(17) $$\begin{array}{r} \Phi^{1}\left(r,\theta ,\varphi \right)=\frac{-p}{4\pi \sigma_{1}r_{z}^{2}}\sin\varphi \overset{\infty }{\underset{n\;=\;1}{\sum }}\left[A_{n}^{1}{\left(\frac{r}{r_{1}}\right)}^{n}+{\left(\frac{r_{z}}{r}\right)}^{n+1}\right]P_{n}^{1}\left(\cos\theta \right)\\ r_{z}<r\leq r_{1} \end{array}$$

(18) $$\begin{array}{r} \Phi^{s}\left(r,\theta ,\varphi \right)=\frac{-p}{4\pi \sigma_{1}r_{z}^{2}}\sin\varphi \overset{\infty }{\underset{n\;=\;1}{\sum }}\left[A_{n}^{s}{\left(\frac{r}{r_{s}}\right)}^{n}+B_{n}^{s}{\left(\frac{r_{s}}{r}\right)}^{n+1}\right]P_{n}^{1}\left(\cos\theta \right)\\ r_{s-1}\leq r\leq r_{s}, \end{array}$$

where φ is the azimuth angle and $P_{n}^{1}$ is the associated Legendre polynomial. When solving for the boundary conditions, Equations (2)–(4), we find that the coefficients $A_{n}^{s}$ and $B_{n}^{s}$ are the same as for the radial dipole solution, see section 2.2.1.

In the results section we compare our analytical solution and the FEM simulations with the two published formulas for the potential in the four-sphere model given in Appendices G and H in Nunez and Srinivasan (2006), and in Appendix A in Srinivasan et al. (1998). For comparison we also present the approximate solution provided in Appendix G.4 in Nunez and Srinivasan (2006). Note that two corrections were done to the model presented in Srinivasan et al. (1998) before comparison. First of all, the multiplication factor *p*/σ_1_ was inserted in Equation (A-1), necessary to give potentials in units of volts. Secondly, a superscript in Equation (A-8) was changed, such that the right-hand-side included $A_{n}^{2}$ instead of $A_{n}^{3}$, since this was obviously a typographical error. For more details on the different descriptions of the analytical four-sphere model, see Appendix 1 in Supplementary Materials.

### 2.3. Finite element method

To find the numerical solution of the four-sphere model we solved the Poisson equation (Equation (1)) using the FEM. The first step was to construct a 3D numerical mesh representing the four-sphere head model geometry. We used the open-source program gmsh (Geuzaine, 2009), optimized using the netgen algorithm (Schöberl, 1997). Figure 1B shows the resulting mesh corresponding to the set of radii listed in Table 1. Note that our 3D FEM model-geometry implementation consists of five spheres: scalp, skull, CSF, and two spheres together representing the brain tissue. However, the two innermost spheres (the innermost having a radius of 6 cm) are set to have the same conductivity, i.e., the value for brain tissue listed in Table 1. Thus, the model is effectively still a four-sphere model. We observed, however, that partitioning the four spheres into five and partitioning the inner sphere to a coarser mesh size reduced the overall mesh size and computational time while retaining the accuracy. The resulting mesh comprised of nearly 12.2 million tetrahedrons (2.1 million odd nodes) and we observed that at this resolution, the numerical results had converged.

The dipole source was treated as two point current sources (Dirac δ functions) and the conductivity was set at each mesh point according to Table 1. The electrodes were modeled as ideal point electrodes. Finally, the Poisson Equation (1) and the Neumann boundary condition, Equation (4), were solved numerically with FEM. All FEM simulations were done with the open-source program FEniCS (Logg et al., 2012; Alnæs et al., 2015), with Lagrange P2 finite elements. The linear systems were solved by the *PETSc Krylov Solver* employed with the *Conjugate Gradient* method, and the *Incomplete LU* factorization preconditioner. In all the cases we tested, the solutions converged in less than 350 iterations when the residual norms were of the order 1e-07.

### 2.4. Software

We provide the Python code to obtain the potentials from a current dipole placed in a four-sphere head model using (i) the analytical formulation and (ii) the numerical method (FEM). This is available under the GNU General Public License version 3 here: https://github.com/Neuroinflab/fourspheremodel. Additionally, the scripts to generate the figures presented in this manuscript are also included. We tested this code in Anaconda Scientific package on a Linux 64 machine. For easy uptake of this resource and verification, we provide the associated conda environment, with all the specific libraries necessary to run this software, and a help file.

## 3. Results

### 3.1. Comparison between analytical and FEM results

**Figure 2:**
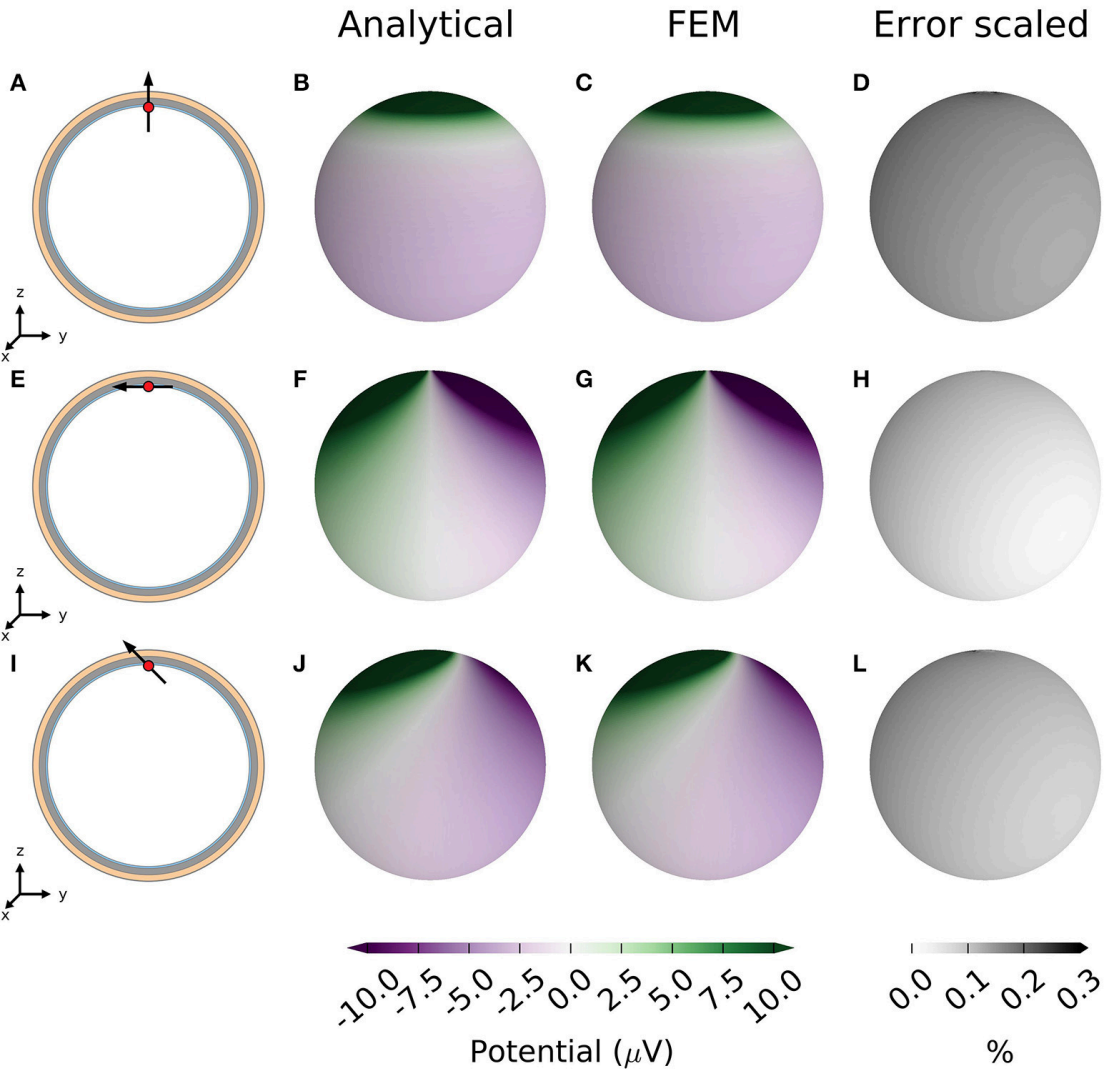
EEG potentials computed with four-sphere model and FEM simulation for radial, tangential, and 45-degree dipole. **(A)** A radial current dipole placed in the brain in the head model as described in Table 1. The dipole (black arrow) is located at **r**_*z*_ = [0, 0, 7.8 cm] (red dot) and has a magnitude 10^−7^ Am to give scalp potentials some tens of microvolts in magnitude, typical for recorded EEG signals. **(B)** Resulting scalp potential calculated with the analytical four-sphere model. **(C)** Scalp potential computed with FEM. **(D)** Absolute difference between results from analytical calculation and FEM, normalized by the global maximum of the magnitude of the potential. The second row, panels **(E–H)** are equivalent to the top row, however for a tangential dipole parallel to the *y*-axis, in the *x* = 0 plane. The bottom row, panels **(I–L)** are equivalent to the top row, however for a dipole that subtends 45 degrees to the *z*-axis in the *x* = 0 plane.

EEG potentials were computed on the scalp surface with the analytical four-sphere model Φ(*r*_4_, θ, ϕ) and compared with the results from the FEM simulations for a current dipole *p*. To mimic a current dipole set up by cortical neurons, a dipole was placed in the brain layer (*s* = 1) of the four-sphere head model, 1 mm below the brain-CSF boundary. We modeled the current dipole to have dipole moment equal to 10^−7^ Am (two point sources of magnitude 100 μA separated by *d* = 1 mm). Three different dipole orientations were tested: a radial dipole parallel to the z-axis, a tangential dipole parallel to the y-axis and a dipole subtending 45 degrees to the *z*-axis in the *x* = 0 plane, cf. Figures 2A,E,I. We found that the analytical and FEM models gave similar results for both radial and tangential dipoles: the absolute value of the difference was more than two orders of magnitude smaller than the computed EEG potential for all dipole orientations (Figure 2). While we show results only for one current dipole in three orthogonal orientations for a single position, the scripts provided are generic and accept arbitrary placement, orientation, and moment of the dipole.

**Figure 3:**
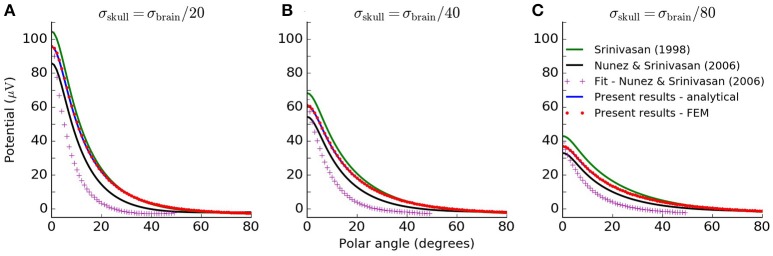
Analytical solution of four-sphere model matches FEM simulation. Scalp potentials from radial current dipole at position *r*_*z*_ = 7.8 cm and magnitude 10^−7^ Am to give results in observable range, while still facilitating direct comparison with the original plots in Srinivasan et al. (1998); Nunez and Srinivasan (2006). The resulting scalp potentials are shown for increasing polar angle θ between the current dipole and the measurement position vector. The different lines show calculations with the various formulations of the four-sphere model discussed in this paper, as well as the FEM simulation. The green line shows potentials obtained from Srinivasan et al. (1998), Appendix A, Equations (A1–11). The black line shows results from applying the formulation given in Nunez and Srinivasan (2006), Appendix G, Equations (G.1.9–10) and (G.2.1–10). The approximate solution from Nunez and Srinivasan (2006), Appendix G.4, Equation (G.4.1–3) is given by the pink crosses. The analytical formulation of the four-sphere model presented here is shown in blue, and the FEM simulation is given by the red dots. Panels **A–C** show results for different values of the skull conductivity, i.e., σ_skull_=σ_brain_/20, σ_brain_/40 and σ_brain_/80, respectively.

A more detailed comparison of EEG potentials predicted by the analytical model and the FEM model is shown in Figure 3. Here the computed EEG signal from a radial current dipole is shown for increasing polar angle θ between the current dipole position vector **r**_*z*_ and the measurement position vector **r**. The sphere radii and conductivity values are consistent with Nunez and Srinivasan (2006) (Table 1). The curve for the analytical results (blue line) overlaps the FEM results (red dots). This figure also demonstrates that previously published formulas give incorrect predictions.

### 3.2. Limiting case

**Figure 4:**
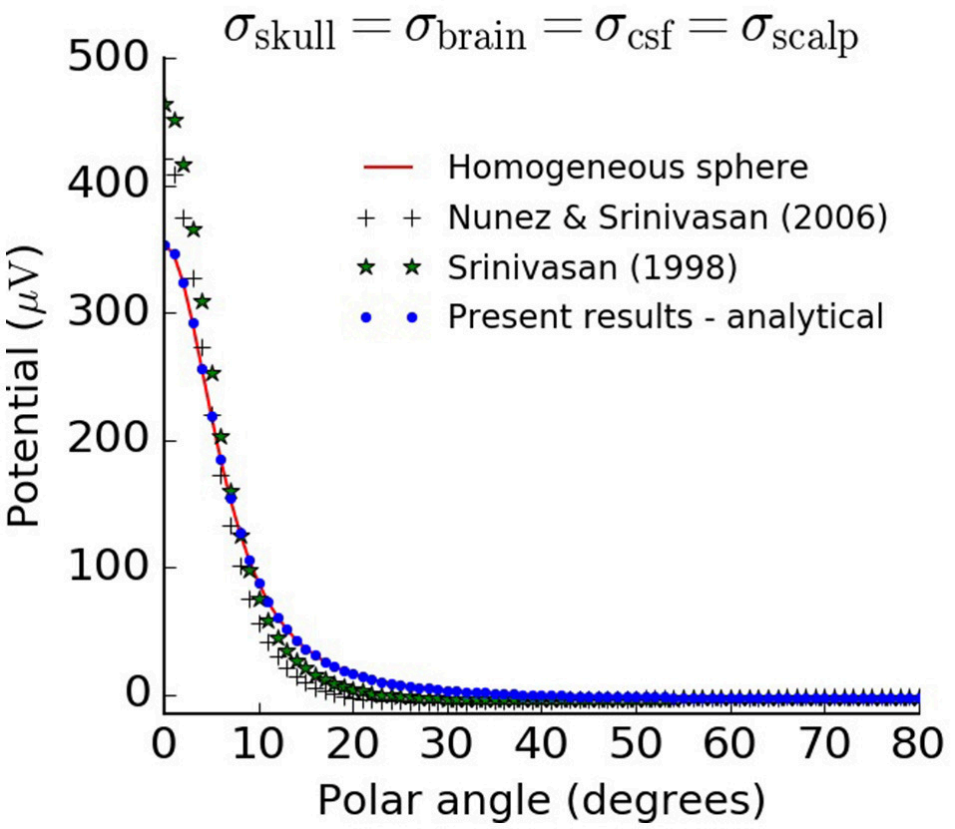
Analytical solution of the four-sphere model satisfies control test for limiting case. Four-sphere model in the limiting case where the conductivity of the skull, CSF, and scalp are equal to the conductivity of the brain, compared to the equivalent model for a single homogeneous sphere, Equation (19). We used a radial dipole of magnitude 10^−7^ Am positioned a distance *r*_*z*_ = 7.8 cm away from the center of the sphere, consistent with Figures 2, 3.

As an additional control we tested the limiting case where the conductivity was set to be the same for all four shells, i.e., σ_brain_ = σ_CSF_ = σ_skull_ = σ_scalp_, and equal to that of the brain (Table 1). In this case, the resulting scalp potentials should be the same as those calculated from a homogeneous single-sphere head model with radius equal to the scalp radius *r*_4_. For a dipole oriented along the radial direction inside a single homogeneous sphere, the surface potentials are given by Equation (6.7) in Nunez and Srinivasan (2006):

(19) $$\begin{array}{l} \Phi (r_{4},\theta )=\frac{p}{4\pi \sigma_{1}r_{4}^{2}}\{\frac{2(\cos\theta -f)}{{\left(1+f^{2}-2f\cos\theta \right)}^{\frac{3}{2}}}\\ \;+\frac{1}{f}\left[\frac{1}{{\left(1+f^{2}-2f\cos\theta \right)}^{\frac{1}{2}}}-1\right]\}, \end{array}$$

where *f* = *r*_*z*_/*r*_4_. Comparison between the simplified four-sphere models and the homogeneous single-sphere model showed perfect agreement for the present formulation, while the formulas listed in Srinivasan et al. (1998) and Nunez and Srinivasan (2006) gave inaccurate predictions (Figure 4).

## 4. Discussion and conclusions

In this note we have revisited the analytical four-sphere model for computing EEG potentials generated by current dipoles in the brain. The main contributions of this paper are the presentation of corrected and validated formulas, as well as the scripts for using them, allowing users to readily apply this important forward model in the field of EEG analysis.

In addition to facilitating the use of the four-sphere model in EEG signal analysis (see, e.g., Wong et al., 2008; Chu et al., 2012; Peraza et al., 2012), the present formulas and scripts will also be a resource for benchmarking comprehensive numerical schemes for computing EEG signals based on detailed head reconstructions using, for example, the FEM (Larson and Bengzon, 2013), or the Boundary Element Method (Brebbia et al., 2012). The FEM approach is not restricted to specific head symmetry assumptions and can take into account an arbitrarily complex spatial distribution of electrical conductivity representing the electrical properties of the head. This is done by constructing a complicated numerical mesh for the head, a task that is often technically challenging. While it is difficult to assure high precision of the given implementation for more complicated and biologically realistic head geometries, the present validated analytical solution for the four-sphere model can serve as one possible ground-truth benchmark. Any FEM or BEM implementation to be trusted, for any analytical model, such as the four-sphere model, should give results in agreement with analytical predictions for different parameter values; here, for example, for various sphere configurations as well as dipole positions and directions. We also provide a set of FEM scripts which model the four-sphere model consistent with the analytical solution.

Forward models with varying complexity are also used to test the accuracy of inverse methods which estimate the dipole source locations from the potentials and electrode positions. All inverse methods are based on a priori assumptions about the volume and conductivity of the brain. Their implementation requires a forward model encoded either as a lead field matrix or otherwise. The analytical solution of the four-sphere head model provides a way to quickly, yet exhaustively, obtain potentials for a wide range of dipole positions. This makes it an attractive option for testing the accuracy of inverse methods.

## Author contributions

SN and TN derived the analytical expressions. CC developed the computational model and did the simulations. AD and GE designed the analytical study. DW designed the computational study. All authors wrote the manuscript.

### Conflict of interest statement

The authors declare that the research was conducted in the absence of any commercial or financial relationships that could be construed as a potential conflict of interest.
