# Supplementary material for: Corrected Four-Sphere Head Model for EEG Signals
Source: Front Hum Neurosci. 2017 Oct 18;11:490. doi: 10.3389/fnhum.2017.00490 (PMC5651266; doi:10.3389/fnhum.2017.00490)
Supplement: Supplementary file 1 [file DataSheet1.pdf]

APPENDIX: MATHEMATICAL DERIVATION OF FOUR-SPHERE MODEL

The four-sphere model equations for radial and tangential dipoles are given in Equations (5), (6), (17) and (18). Here we describe how the seven unknown coefficients (Equation (7)–(16) can be determined by the seven boundary conditions (Equations (2)–(4)). We show the calculations for radial dipoles only, however, the derivation presented applies to both radial and tangential dipoles, due to similarity of the models.

We start by finding the derivative of $\Phi^s(r,\theta)$ from Equation (6):

$$\frac{\partial}{\partial r}\Phi^s(r,\theta) = \frac{p}{4\pi\sigma_1 r_z^2}\sum_{n=1}^{\infty}\left[A_n^s\left(\frac{n}{r}\right)\left(\frac{r}{r_s}\right)^n - B_n^s\left(\frac{n+1}{r}\right)\left(\frac{r_s}{r}\right)^{n+1}\right]nP_n(\cos\theta).$$

For the Neumann boundary condition on the scalp boundary, Equation (4), we make use of the relation above, and get:

$$\frac{\partial}{\partial r}\Phi^4(r_4,\theta) = \frac{p}{4\pi\sigma_1 r_z^2}\sum_{n=1}^{\infty}\left[A_n^4\left(\frac{n}{r_4}\right)\left(\frac{r_4}{r_4}\right)^n - B_n^4\left(\frac{n+1}{r_4}\right)\left(\frac{r_4}{r_4}\right)^{n+1}\right]nP_n(\cos\theta) = 0.$$

$$\Rightarrow A_n^4\left(\frac{n}{r_4}\right) - B_n^4\left(\frac{n+1}{r_4}\right) = 0 \qquad \forall\, n$$

$$\Rightarrow B_n^4 = \frac{n}{n+1}A_n^4. \tag{20}$$

Next, we apply the Dirichlet boundary condition on the skull boundary, i.e., Equation (2) for $s = 3$:

$$\Phi^4(r_3) = \Phi^3(r_3)$$

$$\frac{p}{4\pi\sigma_1 r_z^2}\sum_{n=1}^{\infty}\left[A_n^4\left(\frac{r_3}{r_4}\right)^n + B_n^4\left(\frac{r_4}{r_3}\right)^{n+1}\right]nP_n(\cos\theta) = \frac{p}{4\pi\sigma_1 r_z^2}\sum_{n=1}^{\infty}\left[A_n^3\left(\frac{r_3}{r_3}\right)^n + B_n^3\left(\frac{r_3}{r_3}\right)^{n+1}\right]nP_n(\cos\theta)$$

$$A_n^4\left(\frac{r_3}{r_4}\right)^n + B_n^4\left(\frac{r_4}{r_3}\right)^{n+1} = A_n^3 + B_n^3\,.$$

Inserting the expression for B_n^4, Equation (20), using the notation $r_{ij} \equiv r_i/r_j$:

$$A_n^4\left(r_{34}^n + \frac{n}{n+1}r_{43}^{n+1}\right) = A_n^3 + B_n^3$$

$$\Rightarrow A_n^4 = \frac{n+1}{n}\frac{A_n^3 + B_n^3}{\frac{n+1}{n}r_{34}^n + r_{43}^{n+1}}. \tag{21}$$

Note that the multiplication factor $\frac{n+1}{n}$ is missing in Nunez and Srinivasan (2006), Appendix G, Equation (G.2.9).

Further, we look at the Neumann boundary condition on the skull boundary, i.e. Equation (3) for $s = 3$, using the notation $\sigma_{ij} \equiv \sigma_i/\sigma_j$:

$$\sigma_4 \frac{\partial \Phi^4}{\partial r}(r_3) = \sigma_3 \frac{\partial \Phi^3}{\partial r}(r_3)$$
$$\sigma_4 \left(A_n^4 \frac{n}{r_3} \left(\frac{r_3}{r_4}\right)^n - B_n^4 \frac{n+1}{r_3} \left(\frac{r_4}{r_3}\right)^{n+1} \right) = \sigma_3 \left(A_n^3 \frac{n}{r_3} \left(\frac{r_3}{r_3}\right)^n - B_n^3 \frac{n+1}{r_3} \left(\frac{r_3}{r_3}\right)^{n+1} \right)$$
$$nA_n^4 r_{34}^n - (n+1) B_n^4 r_{43}^{n+1} = \sigma_{34} \left(nA_n^3 - (n+1) B_n^3 \right).$$

Inserting Equation (20),

$$nA_n^4 \left(r_{34}^n - r_{43}^{n+1} \right) = \sigma_{34} \left(nA_n^3 - (n+1) B_n^3 \right),$$

and applying Equation (21),

$$n \, \frac{n+1}{n} \frac{A_n^3 + B_n^3}{\frac{n+1}{n} r_{34}^n + r_{43}^{n+1}} \left(r_{34}^n - r_{43}^{n+1} \right) = \sigma_{34} \left(nA_n^3 - (n+1) B_n^3 \right).$$

From this we find that,

$$B_n^3 = \frac{\frac{n}{n+1}\sigma_{34} - \frac{r_{34}^n - r_{43}^{n+1}}{\frac{n+1}{n} r_{34}^n + r_{43}^{n+1}}}{\sigma_{34} + \frac{r_{34}^n - r_{43}^{n+1}}{\frac{n+1}{n} r_{34}^n + r_{43}^{n+1}}} A_n^3,$$

which we can write as:

$$B_n^3 = V_n A_n^3 \qquad \text{where} \qquad V_n = \frac{\frac{n}{n+1}\sigma_{34} - \frac{r_{34}^n - r_{43}^{n+1}}{\frac{n+1}{n} r_{34}^n + r_{43}^{n+1}}}{\sigma_{34} + \frac{r_{34}^n - r_{43}^{n+1}}{\frac{n+1}{n} r_{34}^n + r_{43}^{n+1}}}. \tag{22}$$

Here, the σ_{34}-term in the numerator of V_n differs from Nunez and Srinivasan (2006) (Equation (G.2.1)) and Srinivasan et al. (1998) (Equation (A-2)) in the sense that the multiplication factor is inverted.

For the CSF Dirichlet boundary condition we can follow the same procedure as for the skull Dirichlet boundary condition, and we get,

$$A_n^3 \left(\frac{r_2}{r_3}\right)^n + B_n^3 \left(\frac{r_3}{r_2}\right)^{n+1} = A_n^2 \left(\frac{r_2}{r_2}\right)^n + B_n^2 \left(\frac{r_2}{r_2}\right)^{n+1}$$
$$\Rightarrow A_n^3 r_{23}^n + B_n^3 r_{32}^{n+1} = A_n^2 + B_n^2.$$

Inserting the expression for B_n^3 from Equation (22):

$$A_n^3\left(r_{23}^n + V_n r_{32}^{n+1}\right) = A_n^2 + B_n^2$$
$$\Rightarrow A_n^3 = \frac{A_n^2 + B_n^2}{r_{23}^n + r_{32}^{n+1} V_n}\ . \tag{23}$$

Here, we notice a typographical error in the expression for A_n^3 in Srinivasan et al. (1998), Equation (A-8): there should be an A_n^2-term in the numerator, not A_n^3.

Next, we apply the Neumann CSF boundary condition. Starting out with,

$$\sigma_3 \frac{\partial \Phi^3}{\partial r}(r_2) = \sigma_2 \frac{\partial \Phi^2}{\partial r}(r_2),$$

and making use of the expressions for B_n^3 and A_n^3, we find that,

$$B_n^2 = Y_n A_n^2 \qquad \text{where } Y_n = \frac{\frac{n}{n+1}\sigma_{23} - \frac{\frac{n}{n+1} r_{23}^n - V_n r_{32}^{n+1}}{r_{23}^n + V_n r_{32}^{n+1}}}{\sigma_{23} + \frac{\frac{n}{n+1} r_{23}^n - V_n r_{32}^{n+1}}{r_{23}^n + V_n r_{32}^{n+1}}}. \tag{24}$$

Note that there's a subtle difference between the Y_n presented here, and Nunez and Srinivasan (2006) (Equation (G.2.2)) and Srinivasan et al. (1998) (Equation (A-3)): The second term of the numerator is a fraction. Here, the r_{23}^n factor should not be multiplied by the whole fraction, but rather only the $\frac{n}{n+1}$-term in the numerator.

The Dirichlet boundary condition on the brain boundary is:

$$\Phi^2(r = r_1) = \Phi^1(r = r_1)$$
$$A_n^2\left(\frac{r_1}{r_2}\right)^n + B_n^2\left(\frac{r_2}{r_1}\right)^{n+1} = A_n^1\left(\frac{r_1}{r_1}\right)^n + \left(\frac{r_z}{r_1}\right)^{n+1}$$
$$A_n^2 r_{12}^n + B_n^2 r_{21}^{n+1} = A_n^1 + r_{z1}^{n+1}.$$

Inserting the expression for B_n^2 from Equation (24):

$$A_n^2\left(r_{12}^n + Y_n r_{21}^{n+1}\right) = A_n^1 + r_{z1}^{n+1}$$
$$\Rightarrow A_n^2 = \frac{A_n^1 + r_{z1}^{n+1}}{r_{12}^n + r_{21}^{n+1} Y_n}. \tag{25}$$

Finally, we solve the Neumann boundary condition on the brain boundary,

$$\sigma_2 \frac{\partial \Phi^2}{\partial r}(r_1) = \sigma_1 \frac{\partial \Phi^1}{\partial r}(r_1).$$

Inserting the expressions for A_n^2 and B_n^2 from Equations (25) and (24), we find,

$$A_n^1 = \frac{\frac{n+1}{n}\sigma_{12} + Z_n}{\sigma_{12} - Z_n} r_{z1}^{n+1} \qquad \text{where } Z_n = \frac{r_{12}^n - \frac{n+1}{n} Y_n r_{21}^{n+1}}{r_{12}^n + Y_n r_{21}^{n+1}}. \tag{26}$$

The A_n^1-term in Srinivasan et al. (1998) (Equation (A-5)) is not consistent with Nunez and Srinivasan (2006) (Equation (G.2.4)) equal to Equation (26): a multiplication factor p/σ_1 is lacking, r_{zl}^{n-1} should be r_{z1}^{n-1}. Moreover, B_n^1 needs to be defined in order for the model description in Srinivasan et al. (1998), Appendix A to give potentials in brain tissue.
