# Supplementary figures and images for: Corrected Four-Sphere Head Model for EEG Signals
Source: Front Hum Neurosci. 2017 Oct 18;11:490. doi: 10.3389/fnhum.2017.00490 (PMC5651266; doi:10.3389/fnhum.2017.00490)

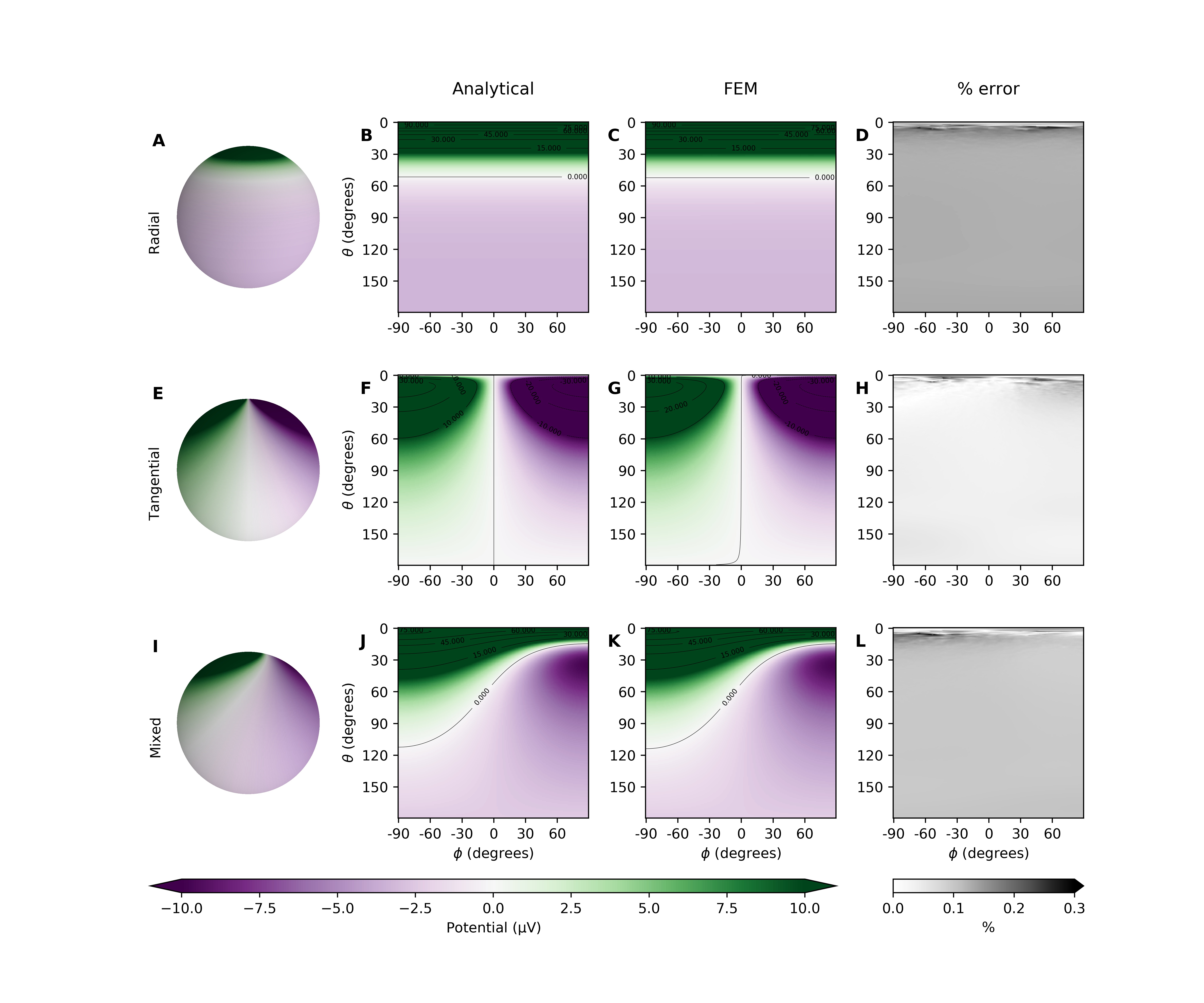

Supplement: Supplementary file 2 [file Image1.jpeg]
